# Interactions between autistics and healthy children and their parents in emotional availability: a comparative study

**DOI:** 10.1017/gmh.2024.102

**Published:** 2024-10-23

**Authors:** Belgin Ustun Gullu, Didem Behice Oztop, Eda Umutlu Aydin, Baris Ors, Merve Cikili Uytun, Esra Yurumez

**Affiliations:** 1 Faculty of Science and Literature, Düzce University, Düzce, Turkey; 2 Department of Child and Adolescent Psychiatry, Ankara University, Ankara, Turkey

**Keywords:** autism, ASD, neurodevelopmental disorders, parenting, emotional availability

## Abstract

In this study, we assessed the interactions of mothers and fathers with their children diagnosed with autism spectrum disorder (ASD) in terms of emotional availability (EA) and compared them with the interactions of healthy controls. Children, aged 13–60 months and applied to the Infant Mental Health Unit between January 2019 and March 2021 and their parents without any clinical diagnosis, were included. The EA levels of mothers and fathers of the autistic group, which included 30 boys and 13 girls, and those of the control group, which included 10 boys and 10 girls, were compared. According to the results obtained, it was determined that the EA levels of mothers and fathers of healthy controls were not different; however, the mothers were more sensitive and better in structuring the content of play compared with the fathers in the ASD group. It was noted that the fathers of children with ASD were more hostile than the mothers. EA should be taken as a criterion to determine the intensity and content of treatment, particularly in ASD. Additionally, increased awareness of fathers in EA may provide better results in the intervention process.

## Graphical abstract

**Figure:**
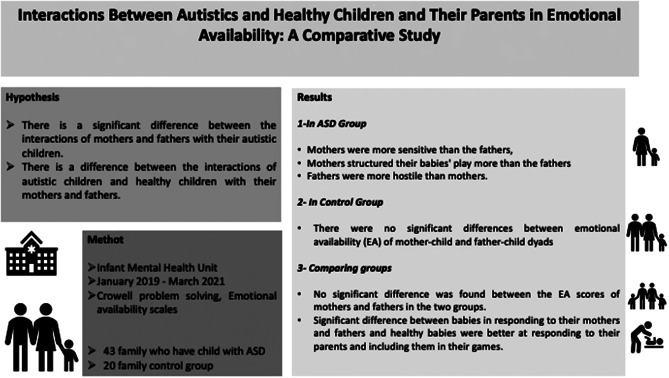

## Impact statement

The early years of life is an important developmental period in which the baby needs the intensive physical and emotional care and attention of both the mother and the father. In some cultures, the mother is primarily responsible for the infant’s care, while the father is responsible for providing for the family financially. Previously, studies have emphasized the importance of the mother–infant relationship, but recent research suggests that the involvement of fathers in their children’s care has significant effects on their development. Emotional availability, which refers to the quality of emotional exchanges between parents and children, is an essential but less studied concept. It focuses on their ability to understand and respond appropriately to each other’s communicative signals. Evaluating the interactions between children with autism and their parents can provide awareness in this field, especially when compared to the findings of healthy children. Our study on father–infant interactions is expected to make important contributions to mental health services and policies in societies where awareness of this issue is less prevalent.

## Introduction

Autism spectrum disorder (ASD) is defined as a neurodevelopmental disorder, the symptoms of which begin in the early stages, and the effects of which continue for life, in which social interaction is limited or absent, and repetitive behaviors (Akın and Düzel, 2023). ASD is classified in DSM-5 into two main groups: social–emotional limitations, nonverbal communication limitations, and difficulties in initiating or maintaining peer relationships. According to the diagnostic criteria, at least two of the following four subgroups must be observed: limited range of interests and repetitive activities; repetitive motor movements, speech, or use of objects; insistence on sameness with a solid aversion to changes in routines or rituals; and an increase or decrease in reactions to sensory aspects of the environment (APA, 2013). According to the results obtained by combining 2020 data, approximately 1 in 36 children has been identified with ASD according to estimates from the CDC’s Autism and Developmental Disabilities Monitoring Network (Maenner et al., 2023).

The most effective and appropriate approach for treating a child with ASD is to provide individual and group special education and to develop their social skills in areas where they might have difficulty (Akçakın, 2001). There are studies indicating that parental involvement and education are essential and effective for treating autistic children (Lord et al., 2018; Oono et al., 2013). The purpose and benefit of having the parents administer the intervention is to increase their awareness and involvement. In the ASD group, emotionally unavailable playing and caregiving affect the parent–infant interactions and infants’ responsiveness to the parents adversely (Gul et al., 2016).

### Emotional availability

Emotional availability (EA) is a relational construct that refers to the quality of emotional exchanges between parents and children and focuses on their reciprocal accessibility and their ability to understand and respond appropriately to each other’s communicative signals (Biringen and Robinson, 1991; Biringen, 2000). The EA construct results from the integration of attachment theory (Ainsworth et al., 1978). Ainsworth et al. (1978) defined emotionally available parents were accessible, responsive, and adjusted to the infant’s signals. Theoretical and empirical evidence about EA showed that parenting behaviors are related to the child’s greater sense of security. It was also shown that higher levels of parental EA are related to secure infant–parent attachment (Bretherton, 2000). Studies using the Emotional Availability Scale (EAS) have investigated EA in parent–child interactions with typically developing children and their mothers (Biringen and Easterbrooks, 2012), but few studies have investigated EA of parents and children with special needs (van IJzendoorn et al., 2007; Gul et al., 2016). Bentenuto et al. (2020) addressed several specific issues regarding the EA of parents of autistic children. Findings showed that mothers and fathers were equally emotionally available to their children. There were no differences between the two in parents’ EA scales or their associations with the child’s level of functioning and severity of symptoms.

### Father–child and mother–child interactions in ASD

Limitations in social communication and interaction, social–emotional areas, nonverbal communication and initiating or maintaining peer relationships in autism also significantly affect the parent–child interaction adversely (El-Ghoroury and Romanczyk, 1999; Ludlow et al., 2012). Additionally, maintaining EA in children with ASD may be particularly challenging because the play themes of the children are often idiosyncratic, and the level of play is usually under the level expected from the child’s age (Dolev, 2009). Parents have essential responsibilities in managing this process. These children face many difficulties, such as surviving and organizing their daily lives, and usually, the greatest burden falls on the mothers (Ören and Aydın, 2020). Besides studies focused on mother–child interaction (Estes et al., 2009), many findings have emphasized that in addition to mothers, fathers’ involvement plays a vital role in the physical, emotional and mental development of children (Fox et al., 1991; Mendonça et al., 2011; Wilson and Durbin, 2013). Especially in recent decades, the social movement involved fatherhood (Amato and Rivera, 1999; Popp and Thomsen, 2017) and recognizing the father’s contribution to child development, especially through play (Bronte-Tinkew et al., 2008), have stimulated a research focus on fathers. Studies conducted recently have revealed that the father’s involvement in early childhood makes significant contributions to the healthy development of the child (Dınız et al., 2021). It was been indicated that the emotional expression of fathers and the interaction and communication between fathers and children have significant predictive effects on the social behaviors of young children (Liu, 2019). Studies have reported that mothers, as primary caregivers, usually take responsibility for providing emotional support and nutrition, creating routines, setting rules and organizing their children (Kellerman and Katz, 1978; Akhan and Batmaz, 2011). On the other hand, Finley et al. (2008) showed that fathers were significantly less involved in all aspects of parental involvement, such as EA and participation, play and the child’s individual needs. According to the results of a study in which father–child and mother–child play interactions were videotaped and coded for parental playfulness, sensitivity, structuring and interventionism, and child negativity, mothers and fathers did not differ in playing games.

In international literature, studies have examined the interaction of fathers with their children as well as that of mothers (Fox et al., 1991; Mendonça et al., 2011; Wilson and Durbin, 2013). There are also studies investigating EA in mother–child and father–child interactions in families with children with ASD (Bentenuto et al., 2020). In Turkey, most studies have investigated mother–child interactions (Töret et al., 2015; Doğan et al., 2016). Therefore, given that father–child interaction is at least as crucial as mother–child interaction in child development and mental well-being (Cooksey and Fondell, 1996; Fagan and Iglesias, 1999) and the limited data on the child–parent interaction with ASD, studies investigating the EA of both mothers and fathers are warranted (Lum and Phares, 2005). Accordingly, this study aimed to investigate father–child compared to mother–child interactions in families with children with ASD by focusing on dyadic EA. The study used observational and retrospective methods to test two hypotheses: first, whether there is a significant difference between the interactions of mothers and fathers with their autistic children. Second, whether there is a difference between the interactions of autistic children and healthy children with their mothers and fathers.

## Materials and methods

### Participants

The sample comprised 63 children who applied to the Infant Mental Health Unit between January 2019 and March 2021 and were diagnosed with ASD according to the DSM-5 criteria. Children having chronic illnesses and global developmental delay regarding to Ankara Developmental Screening Inventory (ADSI) as mentioned below were excluded. The ASD group comprised 13 girls (30.2%) and 30 boys (69.8%) with a mean age of 34.6 ± 11.8 (min.13 to max.60) months, and the control group consisted of 10 (50%) girls and 10 boys (50%) with a mean age of 33.8 ± 6.95 (min.18 to max.47) months.

### Measures

#### Sociodemographic form

This form includes questions about the sociodemographic characteristics of infants and their parents. Questions were asked to the parents during the first application of the families (Table 1).

**Table 1.** Sociodemographic characteristics of groups

	ASD group (*n* = 43) Mean ± SD/*n*(%)	Control group (*n* = 20) Mean ± SD/*n*(%)	*p*
Infant’s age (months)	34.6 ± 11.8	33.8 ± 6.95	.8
(min–max)	(13–60)	(18–47)	
Gender			.12
Female	13 (30.2)	10 (50)	
Male	30 (69.8)	10 (50)	
Mother’s age	34.90 ± 5.53	30.35 ± 4.46	**.002** *
Father’s age	38.73 ± 6.09	33.1 ± 4.2	**<.001** ^**^
Mother’s education/Eeementary sch	3 (7.1)	2 (9.5)	.24
Middle sch	5 (11.9)	1 (5)	
High school	6 (14.3)	7 (35)	
Associate degree	4 (9.5)	2 (10)	
Bachelor’s degree	20 (47.6)	8 (40)	
Master’s/doctorate	4 (9.5)		
Father’s education/elementary sch	2 (4.8)	3 (15)	.36
Middle sch	4 (9.5)	4 (20)	
High school	6 (14.3)	3 (15)	
Associate degree	2 (4.8)	0	
Bachelor’s degree	23 (54.8)	8 (40)	
Master’s/doctorate	5 (11.9)	2 (10)	

*Note*: ASD, autism spectrum disorder; SD, standard deviation.

* *p* < .05.

** *p* < .001.

#### Clinical problem-solving procedure

This is a semi-structured observation procedure designed for children between 24 and 54 months of age to assess caregiver–child interaction and attachment behavior (Crowell and Fleischmann, 1988). Zeanah et al. (1997) extended the procedure for use with children between 12 and 54 months of age. The actual procedure takes about 30–40 min and involves nine consecutive episodes of varying lengths of time, designed to elicit behaviors indicative of some domains of the infant–parent relationship. The episodes are free play (with toys including dolls, cars and a ball, cooking and repair tools), cleanup, playing bubbles, four teaching tasks and separation and reunion episodes, consecutively. Diagnostic Classification of Mental Health and Developmental Disorders of Infancy and Early Childhood, revised edition (DC: 0-3R) (Zero to Three, 2005). The Turkish translation was conducted by Doğan et al. (2005), but it was not published. The Parent–Infant Relationship Global Assessment Scale (PIR-GAS), which categorizes the severity of the relationship disturbance, is included in the DC: 0-3R classification and scored between 90 (well-adapted) and 10 (grossly impaired). In this study, for the observational assessment of parent–child interactions, the Clinical Problem-Solving Procedure was applied in a playroom with one-sided mirror. During observing the stages of the procedure, we used the PIR-GAS scores to assess the association between the quality of the mother–child relationship and the aforementioned variables.

#### Ankara developmental screening inventory

This is a tool based on parental reports and consists of 154 items questioned by a physician to gage the developmental levels of children aged 0–6 years. Language-cognitive, fine and gross motor, social interaction skills and self-care abilities are the subscales assessed. ADSI is a culturally appropriate, valid and reliable developmental instrument (Savasir et al. 1994).

#### Emotional Availability Scale

In this study, parent–child interactions were evaluated using the Infancy/Early Childhood version (3rd edition) of the EAS (Biringen et al. 2000). EAS is a scale that measures parents’ EA and consists of six dimensions. The relationship was evaluated in these six dimensions. Parent and child dimensions are considered separately (Biringen, 2005). The concept of EA is attachment-based and emphasizes the guiding aspect of the parent during the interaction with their child. This shows the supportive presence of the parent against the child’s attempts to explore, assert autonomy and provide clues during interaction (Biringen, 2000). The first studies on EA began with Biringen et al. in 1988. They (Bringen and Robinson, 1991; Bringen et al. 1998; Bringen 2005) developed the ‘EAS’ to determine and evaluate the critical structures for EA in parent–child interaction. The scale is divided into two dimensions, and the assessment includes distinct behavior patterns for the parent and the child. The parent part contains the following sections: sensitivity, structuring, non-intrusiveness and non-hostility; in contrast, child dimensions are evaluated in two parts: the child’s response to the parent and the parents’ involvement in the game led by the child. In the sensitivity sub-dimension, sincere affection, safe communication, mutual enjoyment, warmth, eye contact, relaxing physical touches, understanding the child’s messages and clues, allocating enough time to play, smooth transitions between games and being able to resolve conflicts are expected from the parents. The sensitivity scale rates parents on a scale of 9 (highly sensitive) to 1 (highly insensitive). ***In highly sensitive (9 points),** emotional communication between parent and infant is for the most part positive, appropriate and creative. The highly sensitive parent displays genuine, authentic and congruent interest, pleasure and amusement with the infant. **In generally sensitive (7 points),** this parent is very similar to a ‘9’, but the parent does not interact creatively, although he or she is effectively connected to the infant and interactions are harmonious and enjoyable. **In inconsistently sensitive (5 points),** the parent is sensitive in some ways, but the observer finds it difficult to give this relationship a clean bill of health. Parental inconsistency in behavior may be a telling sign. **In somewhat insensitive (3 points),** insensitivity is typically displayed in one of two general ways: one being an active/harsh style (overly active and over-bearing) and the other being a passive/depressed/effectively flat (noninteractive and silent) style. Still, there are positives here. Both styles suggest unresponsiveness to infant communications and lack many of the features of sensitive interactions described earlier. **In highly insensitive (1 point),** this parent displays few areas of strength in interaction with his/her child.* In the structuring sub-dimension, parents draw a supportive framework to guide the child, establish a game by allowing the child to explore, be an active member of the game, provide necessary information for the child, try structuring the game at a level that will not exceed the child’s expectations and set appropriate rules and limits. The scores for parental structuring range from ‘5’ (optimal structuring) to 1 (nonoptimal structuring). ***In optimal structuring (5 points),** the parent company’s bids are successful in structuring interaction. He or she lets the child lead while providing a supportive frame, that is, the parent offers the child the chance to explore and do things while providing a frame on which the child can build. In the context of limit-setting and discipline, the parent is firm (not harsh) and includes preventive measures whenever possible. **In inconsistent structuring (3 points),** there is overall inconsistency in the parent’s ability to structure and/or set limits. The parent may also show unvarying or repetitive attempts to structure that are not successful. **In non-optimal structuring (1 point),** the parent sets no limits and provides no structure for the child. The parent appears passive, perhaps indulgent. He or she does not provide an adequate scaffold. This parent may engage in parallel play.* The sub-dimension of non-intrusiveness refers to physical intervention, being over-stimulating and not leaving the necessary space for the child, entering the game without the child’s invitation, asking questions too often and determining the direction and course of the game without letting the child go. Parental non-intrusiveness is rated from 5 (nonintrusive) to 1 (intrusive). ***In non-intrusive (5 points),** the parent does not overpower the interactions. He or she lets the child lead and bases play interactions on the child’s lead. The interaction is non-intrusive, smooth and ‘spacious’. The parent is available to the child without being intrusive and has a quality of emotionally ‘being there’ or emotionally available without necessarily doing something to the child. **In somewhat intrusive (3 points),** the parent too frequently sets the pace of the interaction, asking questions, directing the course of play, making suggestions and creating frequent theme changes, as opposed to following the child’s direction. Parental intrusiveness is not striking, however. Such behavior appears more directive and/or slightly overprotective rather than truly intrusive. **In intrusive (1 point),** the parent is highly over-stimulating and does not leave enough space in the interaction for the child to explore and lead. The parent controls the interaction, sometimes even physically, punishing or manhandling, and jumps in to do too much for the child, showing a lack of respect and space for not only the child’s wishes but also abilities. This parent is constantly ‘at’ the child or doing something to the child. This parent appears to want to ‘elicit’ certain behaviors from the child.* In the non-hostility sub-dimension, there are behaviors such as being harsh in the face, voice and expressions, threatening speech or frightening behaviors, harsh behaviors such as hitting the table, teasing or embarrassing, behaviors that show boredom during the game, impatience and increased voice tone. The parental nonhostility scale assesses the degree of hostility, ranging from no observed hostility (‘5’) to covert (‘3’) to overt (‘1’) forms. ***In nonhostile (5 points),** there are no expressions of overt or covert hostility toward the child, as can be discerned by the observer. The general emotional climate appears nonhostile. Slight covert hostility would get a rating of ‘4’. **In covertly hostile (3 points),** although signs of hostility are covert, the parent shows pervasive low-level negative affect, in the form of impatience, discontent, resentment, discomfort, boredom, ‘huffing and puffing’, rolling the eyes, teasing, raising the voice or adopting a long-suffering attitude. **In markedly and overtly hostile (1 point),** this parent is overtly harsh, abrasive and demeaning – facially and/or vocally. Parental behavior is threatening and/or frightening. Threats of separation or threats of abuse may be viewed as very hostile even if the parent is joking about it.* In the child’s response to their parents’ sub-dimension, it is expected that the child will be satisfied with being with his parents, enjoy the interaction, respond frequently and consistently to the parents’ invitation, be open to physical contact and not avoid parents. The child’s responsiveness to the parent is reflected in two aspects of the child’s behavior: (1) eagerness or willingness to engage with the parent following a suggestion or bid for exchange and (2) display of clear signs of pleasure in interaction. The descriptions of the responsiveness rating scale, from 7 (optimal) to 1 (clearly nonoptimal) were defined. ***In optimal in responsiveness (7 points),** this child shows an optimal balance between responsiveness to the parent and autonomous activities; such behavior is combined with an affectively positive stance. He or she often responds to the parent’s bids, but without any sense of urgency or necessity. The child generally shows pleasure and eagerness in attending to the parent’s comments, suggestions, questions and demonstrations. **In moderately optimal responsiveness (5 points),** the child is still affectively positive and responsive (overall) but is less so or there is a slightly more blasé or just OK quality. The child has a generally responsive quality but again it is just OK, rather than the beautiful, spectacular sort seen in a ‘7’. Child responsiveness just slightly under the spectacular quality should receive a ‘6’. **In somewhat nonoptimal responsiveness (3 points),** a rating hovering around ‘3 ‘should be given whenever there are serious concerns about the child’s emotional and behavioral responsiveness toward the parent. A ‘3’ refers to emotional and behavioral responsiveness indicative of nonoptimal interactions in the dyadic relationship. Not only does the child not show a good balance between autonomous pursuits and responsive behaviors toward the parent, but he or she is less happy, content and/or emotionally robust. **In clearly nonoptimal responsiveness (1 point),** this child rarely shows emotional and behavioral responsiveness (of the optimal kind) when engaged with the parent and rarely responds to a parental initiative. The balance of autonomous pursuits and responsive behaviors is clearly not optimal; further, there are serious concerns about this child’s emotional health.* In the sub-dimension of involving the parents in the game, the child is expected to establish a balance between playing the game independently and involving the parents in the game, which involes initiating interaction, inviting the parents to the game, telling the parents about the game, asking questions and establishing eye contact. The child involvement scale assesses the degree to which the child attends to and engages the parent in play. The scale ranges from 7 (optimal) to 1 (nonoptimal). ***In optimal involving behaviors (7 points),** this child shows a balanced pattern between autonomous play and drawing the parent into interaction. He or she tries to engage the parent in the interaction and appears eager to do so in a nonanxious wa*y. *The child seems interested in engaging the parent in interaction without compromising autonomous pursuits. **In moderately optimal involving behaviors (5 points),** this child shows more interest in the task at hand than in involving behaviors. The child is much more oriented toward being alone or playing alone than in engaging in interaction with the parent. There is a periodic request of the parent’s attention and interest. **In somewhat nonoptimal in involving behaviors (3 points),** this child does not show a style of optimally drawing the parent into play or interaction, that is, by showing a balance between involving behaviors and pursuits. **In clearly nonoptimal in involving behaviors (1 point),** this child does not optimally orient toward the parent. He or she does not show a good balance between involving behaviors and autonomous pursuits at all* (Biringen, 2005). This scale has no cutoff point. The coherence of EA dimensions has been reported to be approximately .80 (Biringen, 2000). Our clinical team consists of experts who participated in EAS training given by Zeynep Biringen. Inter-rater reliability (based on three participants) was established between each of the three coders, and the correlation ranged from 0.91 to 0.99.

### Procedure

The children who applied to the Infant Mental Health Unit with their parents (both mother and father) were included in the study. The psychiatric examinations were conducted by a child and adolescent psychiatrist, and infants diagnosed with ASD according to the DSM-5 criteria participated in the study. The developmental stage was measured using ADSI. The children who were diagnosed with ASD according to the DSM-5 criteria and did not show comorbid global developmental delay according to ADSI comprised the ASD group of the current study. The children who had no psychiatric or neurodevelopmental diagnosis according to DSM-5 criteria and who were in the typical developmental range according to ADSI comprised the control group. The procedures of the Clinical Problem-Solving Procedure were explained to the parents, and verbal and written consent were obtained. The parents who agreed to participate in our study were then invited to complete sociodemographic forms and were observed with their children in our playroom with a one-sided mirror. Each of the presented episodes was conducted in order, and these episodes were observed by trained raters. After the administration of CPSP, the clinical formulation was recorded with the consensus of a group of professionals, based on DC: 0-3R. The research protocol was approved by the local ethical committee of the XXX University Faculty of Medicine (Approval No. 2022/320). This study follows the Declaration of Helsinki. The data obtained are available and can be provided by researchers in case of need. This study was not preregistered.

### Statistical analysis

Data analysis was performed using the SPSS 26.0 package program, and *p* < 0.05 values were considered statistically significant. Shapiro–Wilk test was used to analyze the homogeneity of variables. Among-group differences in demographic variables were analyzed using Mann–Whitney U test for non-homogenous variables. Associations between categorical variables were examined using Chi-Square or Fisher Exact analysis. The paired sample *t*-test was used to compare mothers’ and fathers’ EAS scores for each group. Among-group differences in EAS scores were analyzed using the independent sample *t*-test.

It was calculated as 20 patients for each group according to the independent sample *t*-test, sufficient to determine the difference between the groups at the 0.05 significance (α) level with 80% power, which was calculated with the G-Power program.

## Results

### Sociodemographic variables of the ASD and control groups

Sociodemographic variables are shown in Table 1. It was found that the mean of the mother’s age and the mean of the father’s age were significantly higher in the ASD group than in the control group. There was no significant difference between the age and gender distributions of the two groups (*p* > 0.05).

### Comparison of the EA scales in mother–child and father–child dyads

The parents of infants diagnosed with autism were compared in terms of EA sub-dimensions, and the results are shown in Table 2. According to the results obtained, it was determined that the mothers were more sensitive than the fathers, and the mothers structured their babies’ play more than the fathers. It was noted that fathers were more hostile than mothers. No significant difference was found between the behaviors of the children in terms of involving their parents in the game and their responses to the parents.

**Table 2.** Comparison of EAS scores of mothers and fathers for both groups

	ASD group (*n* = 43)		Control group (*n* = 20)		
	M	SD	M	SD	*p*
Msensitivity	4.79	1.85	5.22	1.21	.83
Fsensitivity	4.39	1.83	5.11	1.45	.32
Mstructuring	2.54	1.03	2.69	.85	.7
Fstructuring	2.29	1.01	2.38	.93	.76
Mintrusiveness	4.04	.99	3.88	1.13	.65
Fintrusiveness	3.88	1.07	4.05	1.06	.73
Mhostility	4.74	.72	4.66	.68	.57
Fhostility	4.88	.39	4.94	.23	.54
Mchild res.	2.83	1.29	4.86	1.39	**<.001** ^**^
Fchild res.	2.68	1.24	4.94	1.39	**<.001** ^**^
Minvolvement	2.52	1.40	4.75	1.37	**<.001** ^**^
Finvolvement	2.32	1.35	4.61	1.33	**<.001** ^**^
PIRGAS	50.23	14.05	68	13.6	**<.001** ^**^

*Note*: ASD, autism spectrum disorders; EAS: emotional availability; F, father; M, mother.

** *p* < .001.

The parents of the control group were also compared in terms of EA sub-dimensions, and the results are shown in Table 3. There were no significant differences between EA of mother–child and father–child dyads in the control group.

**Table 3.** Emotional availability comparison of mothers and fathers of infants diagnosed with autism

	*M*	SD	*p*
Msensitivity	4.79	1.85	**.03** *
Fsensitivity	4.39	1.83	
Mstructuring	2.54	1.03	**.02** *
Fstructuring	2.29	1.01	
Mintrusiveness	4.04	.99	.3
Fintrusiveness	3.88	1.07	
Mhostility	4.74	.72	**.05** *
Fhostility	4.88	.39	
Mchild res	2.83	1.29	.19
Fchild res	2.68	1.24	
Minvolvement	2.52	1.40	.15
Finvolvement	2.32	1.35	

*Note*: F, father; M, mother. Paired sample *t*-test.

* *p* < .05.

### Comparison of the EA scales in mother–child and father–child dyads for both groups

Mothers and fathers of infants diagnosed with autism were compared with mothers and fathers of healthy infants in terms of EA (Table 4). According to the results obtained, no significant difference was found between the sensitivity, structuring, non-intrusiveness and non-hostility sub-dimensions of mothers in the two groups. Similarly, no difference was found between the fathers in terms of those sub-dimensions of EA. It was noted that there was a significant difference between babies with and without autism in responding to their mothers and fathers and involving them in the play. Healthy babies were better at responding to their parents and including them in their games.

**Table 4.** Emotional availability comparison of mothers and fathers of the control group

	*M*	SD	*p*
Msensitivity	5.22	1.21	.9
Fsensitivity	5.11	1.45	
Mstructuring	2.69	.85	.13
Fstructuring	2.38	.93	
Mintrusiveness	3.88	1.13	.77
Fintrusiveness	4.05	1.06	
Mhostility	4.66	.68	.13
Fhostility	4.94	.23	
Mchild res.	4.86	1.39	.39
Fchild res.	4.94	1.39	
Minvolvement	4.75	1.37	.72
Finvolvement	4.61	1.33	

*Note*: F, father; M, mother. Paired sample *t*-test.

## Discussion

In the current study, considering the lack of knowledge and research on the EA of fathers in our country, we needed to draw attention to the interaction of fathers with their babies diagnosed with autism, which is an increasingly common neurodevelopmental disorder.

Forty-three infants with ASD and, 20 infants without ASD, and their parents who applied to the Child and Adolescent Psychiatry Department of the XXX University between January 2019 and March 2021 were evaluated in the Infant Mental Health Unit.

Regardless of whether they have a diagnosis, mothers are perceived as primarily responsible for the care of babies in our society. In addition to providing basic care, meeting the baby’s emotional needs and playing with him are expected from the mother. In our cultural context, studies are mostly interested in the evaluation of mothers rather than both parents in baby care (Yıldız, 2008; Şayık and Örsal, 2019). Recently, with the increasing involvement of fathers in the care of their babies, studies on the communication, interaction and emotional accessibility of babies with their fathers have increased (Day and Lamb, 2004; Liu, 2019). To the best of our knowledge, this is the first study in Turkey to examine the EA of mothers and fathers of infants diagnosed with ASD and to compare the EA of mothers and fathers of infants with typical development. Additionally, the number of studies investigating fathers’ participation in EA is still limited (Lovas, 2005).

In this study, mothers and fathers of infants diagnosed with ASD were compared in terms of EA. Although there was no difference between the EAS of mothers and parents in the control group, mothers of infants diagnosed with autism were found to be more sensitive than fathers. In the ASD group, mothers structured their infants’ play more than fathers. In a qualitative study by Oğuz and Sönmez (2018) in our country, it was found that mothers of children with ASD exhibit a more sensitive–responsive interaction than the fathers. In another similar study conducted with families with ASD children, it was shown that mothers have more sensitive and more responsive interactions with their children compared with the fathers (Karaaslan, 2016). These findings are consistent with the results of the current study. Similar to that, other studies indicated that fathers, compared to mothers, may use less parentese (i.e., ‘baby talk’), may speak less frequently to infants who later develop ASD (Cohen et al. 2013) and may be less verbally responsive to their children (Flippin and Watson, 2015). In the literature, different results have attracted attention. Bentenuto et al. (2020) addressed several specific issues regarding the EA of parents of children diagnosed with ASD. Findings showed that mothers and fathers were equally emotionally available to their children. There were no differences between the two groups in parents’ EA scales or their associations with child’s level of functioning and severity of symptoms.

In addition, it is known that Turkey sees a bridge between East and West (Greaves, 2007). Turkey could not remove the Eastern culture in its efforts to westernize and remained in the east–west trap (Berber, 2012). The difference between East and West cultures becomes more evident, especially regarding child care. While activities such as household chores and child care are still prominent for women, work roles are becoming more important than family roles for men (Savaş, 2018). In Turkey, mothers undertake more parenting responsibilities than fathers. These gender roles of mothers and fathers in Turkey also support the results of our study.

An important finding of the current study is that fathers of babies with autism are more hostile than mothers. In this study, to evaluate the EA levels of the parents, the playing process of the mother, father and baby was observed. It was also checked whether the child responded to the messages and play attempts from the parents and, whether he followed the instructions. It was observed that the parents were more inclined to introduce the toys during play. They also expected their babies to say the toys’ names and use the toys in accordance with their functions. In this respect, as secondary caregivers and attachment objects, it may be comprehensible for fathers to display a more hostile attitude than mothers when the infants do not respond to them. It is thought that fathers may be more impatient and show harsh attitudes and behaviors toward their babies to whom they do not receive a response. In a qualitative study (Bıçak, 2009) including the mothers of children diagnosed with ASD, it was found that mothers could accept and adjust to the diagnoses more easily than fathers. In a study by Aslan (2020), it was noted that in the case of a new diagnosis, mothers tend to use coping methods such as social–emotional support, and fathers are more likely to deny the diagnosis. Consistent with these results, according to the Parental Acceptance Rejection Theory; notably in cases of parental rejection, harsh, hostile and distant attitudes and behaviors are exhibited toward the child (Rohner, 2006; cited in Gülay, 2011). There has been no study comparing the acceptance and rejection behaviors of mothers and fathers who have children with ASD. Research has mainly focused on mothers’ acceptance–rejection behaviors (Kaytez and Durualp, 2016). In this study, it was observed that the mothers, also the primary caregivers, showed more accepting behaviors in their relations with their children (more sensitive to their children, structuring their plays), while the fathers exhibited more rejecting behaviors (hostile attitudes; being harsh in the face, voice and expressions; threatening toward their children; talking or indicating frightening behaviors; harsh behaviors such as hitting the table, teasing or embarrassing; behaviors indicating boredom during the play; impatience and raising the tone of voice).

The mothers and fathers of the two groups were compared, and it was indicated that, in terms of sensitivity, structuring, divergence and hostility sub-dimensions, the mothers or fathers of infants with and without ASD were not different. Although the fathers of infants diagnosed with autism were more hostile than the mothers, another striking finding was that there was no difference between the fathers of ASD and healthy infants. This might be related to the small sample size of the current study. On the other hand, parents of a child with ASD have to show additional effort (Bitsika et al., 2013) and may experience many difficulties due to limitations in social communication and interaction, social–emotional areas and nonverbal communication (El-Ghoroury and Romanczyk, 1999; Ludlow et al., 2012). In spite of the effort to get involved, not being able to manage to engage with the child and confronting with child’s challenging behavior may affect the father’s parental satisfaction and self-confidence (Argumedes et al., 2018). Therefore, due to not receiving any answer, fathers may become hostile to their children.

Additionally, it was found that compared with the children without autism, children with autism responded less to their mothers and fathers and included them less in the play. In longitudinal studies examining the social life of families with autistic children, it was recognized that the significant deficiencies of children in social relations, communication and reciprocity made it difficult for parents to interact with their children at a responsive level (Gray, 2002; Leach and Rocque, 2011). In a study conducted in our country, infants diagnosed with autism showed lower response and attention to their mothers than infants diagnosed with developmental delay and other psychiatric disorders (Gul et al., 2016). Developmental delays in motor and nonverbal areas, verbal and motor imitation, inadequacies in playing symbolic games and mutual play, as well as in social and communication areas in children with autism cause them to respond less to the individuals they interact with, and their parents may participate less in their plays or activities. Ultimately, this may be related to the fact that ASD is a disorder that negatively affects and complicates communication and relationship quality (Dissanayake and Crossley, 1997; Zwaigenbaum et al., 2005; Ludlow et al., 2012; Wilke et al., 2012).

### Limitations

The most important limitation of our study is the inclusion of a control group from the clinical sample. However, because of the COVID-19 pandemic, a control group could not be chosen from the community sample and we had to conduct the study retrospectively. In addition, the effects of some factors, including symptom severity and parental age, were not considered in the comparisons of the groups. A more extensive sample with participants matched in gender and number would increase the generalizability of the study. On the other hand, for future studies, measuring parents’ stress, anxiety and depression levels and controlling the time elapsed since diagnosis will make significant contributions to the field.

The clinical results of these findings highlight the importance of analyzing the EA levels of infants diagnosed with autism in both mother–infant and father–infant interactions. It is thought that adopting an intervention program suitable for the EA in ASD, which is a disorder that needs to be intervened early, will contribute to the course of treatment. It is a matter of debate in the literature whether EA should be taken as a criterion to determine the intensity and content of treatment (Wiefel et al., 2005). In addition, it is thought that increased awareness of fathers in EA may provide better results in the intervention process.

## Data Availability

The datasets used and/or analyzed during the current study are available from the corresponding author upon reasonable request.
