# Supplementary material for: Interactions between autistics and healthy children and their parents in emotional availability: a comparative study
Source: Glob Ment Health (Camb). 2024 Oct 23;11:e93. doi: 10.1017/gmh.2024.102 (PMC11505039; doi:10.1017/gmh.2024.102)
Supplement: Peer review history

# Editor report: Recommendation: Interactions between autistics and healthy children and their parents in emotional availability: a comparative study — R0/PR2

**DOI:** 10.1017/gmh.2024.102.pr2

**Affiliations:** Psychiatry, Makerere University CHS, Uganda

Dear Authors,

Please , in addition to the review comments, address the following issues:

1) Make the title more informative, by adding where the study was carried out and methods e.g a comparative study, a retrospective study etc

2) Please your paper needs English editing e.g could you be meaning ‘according to DSM’ and not regarding DSM?

# Editor report: Decision: Interactions between autistics and healthy children and their parents in emotional availability: a comparative study — R0/PR3

**DOI:** 10.1017/gmh.2024.102.pr3

**Affiliations:** Johns Hopkins University Bloomberg School of Public Health, United States

No accompanying comment.

# Editor report: Recommendation: Interactions between autistics and healthy children and their parents in emotional availability: a comparative study — R1/PR5

**DOI:** 10.1017/gmh.2024.102.pr5

**Affiliations:** Psychiatry, Makerere University CHS, Uganda

No accompanying comment.

# Editor report: Decision: Interactions between autistics and healthy children and their parents in emotional availability: a comparative study — R1/PR6

**DOI:** 10.1017/gmh.2024.102.pr6

**Affiliations:** Johns Hopkins University Bloomberg School of Public Health, United States

No accompanying comment.
